# Inflammasome components ASC and AIM2 modulate the acute phase of biomaterial implant-induced foreign body responses

**DOI:** 10.1038/srep20635

**Published:** 2016-02-10

**Authors:** Susan N. Christo, Kerrilyn R. Diener, Jim Manavis, Michele A. Grimbaldeston, Akash Bachhuka, Krasimir Vasilev, John D. Hayball

**Affiliations:** 1 Experimental Therapeutics Laboratory, Sansom Institute and Hanson Institute, School of Pharmacy and Medical Science, University of South Australia, Adelaide, SA, 5000, Australia; 2 Robinson Research Institute, School of Paediatrics and Reproductive Health, University of Adelaide, Adelaide, SA, 5005, Australia; 3 Centre for Neurological Diseases, SA Pathology, Adelaide, SA 5000, Australia; 4 Centre for Cancer Biology, University of South Australia and SA Pathology, SA 5000, Australia; 5 Mawson Institute, University of South Australia, Adelaide, SA 5095, Australia; 6 School of Medicine, University of Adelaide, Adelaide, SA 5005, Australia

## Abstract

Detailing the inflammatory mechanisms of biomaterial-implant induced foreign body responses (FBR) has implications for revealing targetable pathways that may reduce leukocyte activation and fibrotic encapsulation of the implant. We have adapted a model of poly(methylmethacrylate) (PMMA) bead injection to perform an assessment of the mechanistic role of the ASC-dependent inflammasome in this process. We first demonstrate that ASC^−/−^ mice subjected to PMMA bead injections had reduced cell infiltration and altered collagen deposition, suggesting a role for the inflammasome in the FBR. We next investigated the NLRP3 and AIM2 sensors because of their known contributions in recognising damaged and apoptotic cells. We found that NLRP3 was dispensable for the fibrotic encapsulation; however AIM2 expression influenced leukocyte infiltration and controlled collagen deposition, suggesting a previously unexplored link between AIM2 and biomaterial-induced FBR.

The inflammasome is a multiprotein complex that regulates the release of potent IL-1β and IL-18 cytokines in a broad range of inflammatory situations^1^. Triggered sensor proteins recruit apoptosis-associated speck-like protein containing CARD (ASC), and pro-caspase-1 to allow self-activation into caspase-1 for cleavage of pro-IL-1β and pro-IL-18 into their active forms, IL-1β and IL-18, respectively^2^. The plasticity of inflammasome triggers is evident in the growing body of evidence implicating inflammasome activation during biomaterial implantation due to the associated cell damage that may be caused during surgical implantation and subsequent host reactions.

The use of biomaterials is an ever-expanding industry aimed at repairing, replacing or enhancing biological tissues with materials that have been fabricated in a controlled and reproducible manner. However, the function of biomaterial implants and devices can be compromised by the development of a foreign body response (FBR), an acute sterile innate immune inflammatory reaction which overlaps with tissue vascularisation and remodelling, and ultimately fibrotic encapsulation^3^. Immediate blood protein adsorption onto the biomaterial surface directs the subsequent acute inflammation, mediated by frontline neutrophils and monocyte/macrophages^4^ secreting pro-inflammatory cytokines that facilitate further monocyte/macrophage recruitment, activation and fusion resulting in the formation of foreign body giant cells (FBGCs)^5^,^6^. The release of various reactive oxygen and nitrogen species, degradative enzymes and acids by FBGCs can directly facilitate biomaterial degradation and implant failure and this phase also marks the transition to a chronic inflammatory state, associated with vascularisation and tissue remodelling.

Despite the well-described cellular pathways of the FBR, the molecular regulators and mechanisms that drive innate cell responses remain to be solved. Therefore, a key area of molecular investigation is the potential role of the inflammasome in biomaterial-induced FBR, in particular the NLRP3 inflammasome because of its activation by non-phagocytosable particles, such as asbestos and silica^7^, and nanodebris typically derived from implants^8^,^9^. Despite the understanding of inflammasome-independent pathways of IL-1β release, the involvement of the inflammasome has also been implicated for macroscopic biomaterials that cannot be phagocytosed, or do not generate wear debris or particulates. This is based on reports of IL-1β detection at the local implant site *in vivo*, and IL-1β secretion by biomaterial-adherent macrophages *in vitro*^10^,^11^. Recently, Malik *et al.* (2011) were the first to demonstrate the direct involvement of ASC, caspase-1 and NLRP3, in controlling leukocyte recruitment within the first 24 h upon PMMA bead injection^12^.

Therefore, the aim of this study was to investigate the role of the inflammasome in the initiation and progression of the FBR by injecting macro-sized (125–180 μM) PMMA beads into the peritoneum of mice. The immunophenotype of cell infiltration, PMMA bead aggregation, serum protein and cell-mediated protein deposition was quantified at various time points to encompass the dynamic and temporal kinetics of the bead-induced FBR. This model was then used to assess the role of ASC on the FBR because it is the common mediator amongst the inflammasomes. In the absence of ASC, we observed that cell infiltration and collagen deposition was altered, but the corresponding sensor protein NLRP3 was dispensable for macrophage recruitment during the acute and chronic phases of the FBR. Therefore, we hypothesised that the absent in melanoma 2 (AIM2) inflammasome, which binds double stranded (ds) DNA from apoptotic cells or mitochondrial DNA following host cell disruption, may be involved in the FBR. Comprehensive profiling of inflammatory cells and proteins revealed a potential role for the ASC-dependent inflammasome in biomaterial-induced FBR as IL-1β was reduced in ASC^−/−^ and AIM2^−/−^ mice, and delayed in NLRP3^−/−^ mice when compared to wild-type mice. Furthermore, our findings revealed a potential inflammasome-independent role for the AIM2 sensor protein based on the premature collagen deposition and high concentrations of pro-fibrotic transforming growth factor (TGF)-β1, which was not observed for ASC^−/−^ mice. Hence, this is the first study to provide a detailed account of leukocyte recruitment and cytokine profiles during the FBR, including an assessment of IL-1β and IL-18 levels, and to report on a role for AIM2 in the FBR.

## Results

### The injection of PMMA beads generates an inflammatory response that resembles the acute phase of the foreign body response

**Figure 1:**
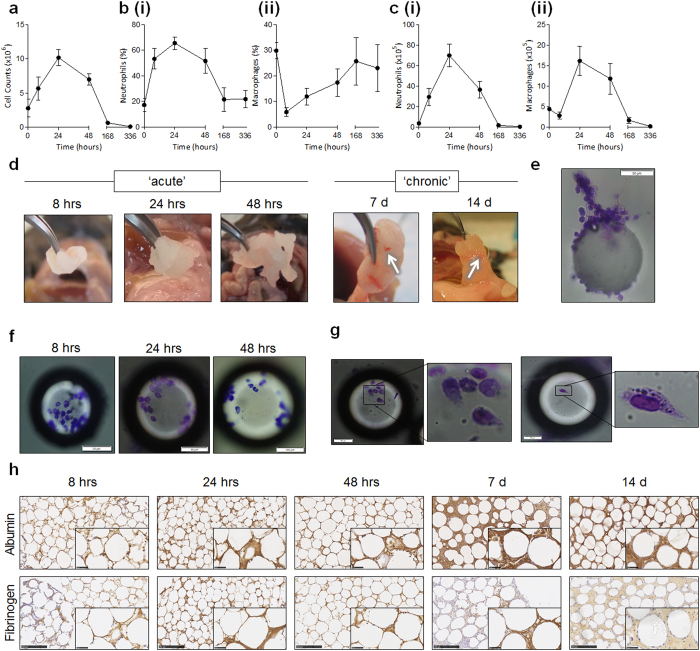
The injection of PMMA beads generates an inflammatory response that resembles the acute phase of the foreign body response. PMMA beads (125–180 μm, 200 μg/mouse) were injected i.p and (**a**) peritoneal lavages were performed for the detection of total cell numbers in the ‘acute’ phase of 8 h, 24 h, or 48 h, and the ‘chronic’ phase of 7 days or 14 days within wild-type B6 mice. (**b**) The proportion of (ii) neutrophils (CD11b^+^ Gr-1^+^) and (ii) macrophages (CD11b^+^ F4-80^+^) were quantified in peritoneal exudates using flow cytometry. The 0 hr control time point is representative of mice injected with diluent as a comparator of homeostatic cellular phenotype. (**c**) To represent accurate changes in cell populations, absolute cell numbers for (i) neutrophils and (ii) macrophages were calculated. (**d**) PMMA beads that were injected as loose particles formed solid aggregates *in vivo* that could be surgically removed. (**e**) Inspection of individual beads retrieved from the peritoneal cavity revealed the development of a proteinous matrix (scale bar, 50 μm), and (**f**) the direct binding of leukocytes to the beads as detected using Diff-Quik staining (scale bar, 50 μm except for 48 hrs which represents 100 μm). (**g**) Adherent cells appeared to alter cytoskeletal morphology when bound to individual beads (scale bar, 50 μm). (**h**) PMMA bead aggregates were sectioned at 5 μm for immunohistochemistry staining of albumin and fibrinogen as observed by brown staining. Scale bar, 250 μm. n = 4–8 mice/group. The mean ± SEM are shown. P values were calculated and no significance was detected as deduced via two-way analysis of variance.

To assess the role of the inflammasome in the FBR, we adapted an *in vivo* model of biomaterial-induced inflammation using peritoneal injections of PMMA beads^12^ to generate events of the FBR. When PMMA beads were injected into the peritoneal cavity of B6 (wild-type) mice, an increase in peritoneal exudate cell numbers from 8 h post-injection was observed, which peaked at 24 h with a total of 10.2 ± 1.2 × 10^6^ cells, before a steady decline (Fig. 1a). Phenotypic analysis of peritoneal exudates (Fig. 1b) revealed the predominant cell type was neutrophils, which increased in a similar pattern to total cell infiltration, and represented 65.5% of the population at 24 h (Fig. 1b(i)). In contrast, the proportion of macrophages in the peritoneal exudate declined from 29.8% to 5.5% of the population within the first 8 h, before they recovered to homeostatic values by day 7 (Fig. 1b(ii)). However, to account for the increase in total cell numbers within the peritoneal cavity, the kinetics of cell recruitment was more accurately represented as an absolute number because it is a product of both percentages and total cell counts (Fig. 1c). These results showed that both neutrophils (Fig. 1c(i)) and macrophages (Fig. 1c(ii)) were actively recruited to the peritoneal cavity and peaked at 24 h with a total of 69.8 ± 11 × 10^5^ cells and 16.1 ± 3.6 × 10^5^ cells, respectively.

When the peritoneal cavity was surgically dissected, the injected PMMA beads were found to have aggregated *in vivo*, and this was evident from as early as 8 h (Fig. 1d). In the acute phase representing 8–48 h, the aggregated bead clump was soft and white in colour. In the chronic phase (day 7 and day 14), aggregates were yellow, more solid, and appeared to have become vascularised as blood vessels (white arrows) were observed within the bead aggregate (Fig. 1d). Disaggregated beads stained with Diff Quik were observed to have initiated a proteinous network (Fig. 1e), and demonstrated adherent leukocytes interacting with individual beads throughout the acute phase of the response (Fig. 1f). A closer inspection of adherent cells revealed altered cell morphology, potentially due to the curvature of the beads, and in some cases, the cells appeared to be migrating as evidenced by the leading edge morphology (Fig. 1g).

Next, the protein composition of the bead aggregates were assessed using immunohistochemistry (IHC) for qualitative analysis of albumin and fibrinogen (Fig. 1h). Albumin and fibrinogen were detected as early as 8 h, as indicated by the dark brown staining amongst the beads, and these serum proteins were observed throughout the inflammatory period (Fig. 1h). Therefore, the adsorption of albumin and fibrinogen, in addition to large cell infiltrate and recruitment of neutrophils and macrophages are consistent with the acute phase of the FBR.

### Injected PMMA beads undergo fibrotic encapsulation and vascularisation that resembles the chronic phase of the foreign body response

**Figure 2:**
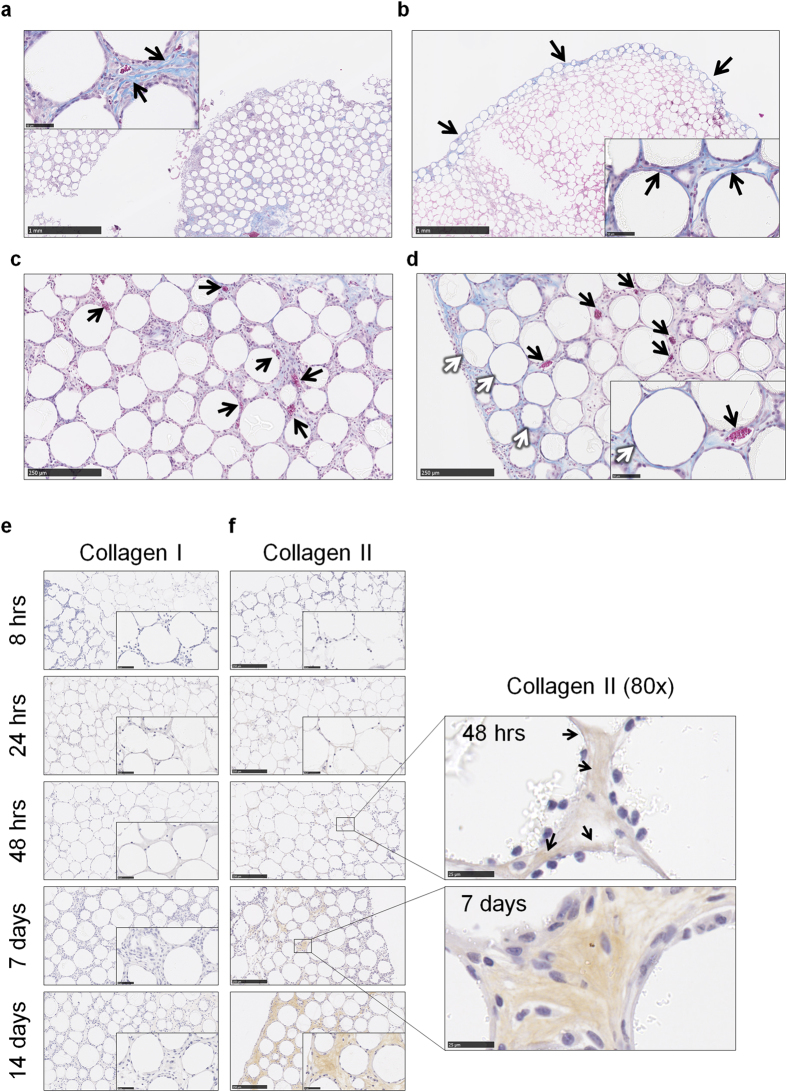
Injected PMMA beads undergo fibrotic encapsulation and vascularisation that resembles the chronic phase of the foreign body response. PMMA bead aggregates from wild-type B6 mice at (**a**) day 7 or (**b**) day 14 were sectioned at 5 μm and stained with Masson’s Trichrome for the detection of pan collagen which is stained blue, and nuclei stained dark purple. (**c**) At day 7 and (**d**) day 14 red blood cells that stained red were observed in the blood vessels (black arrows) that had formed within the bead aggregates. (**d**) We also observed encapsulation of individual beads by collagen (stained blue) as highlighted by the white arrows. PMMA bead aggregates were sectioned at 5 μm for immunohistochemistry staining of (**e**) collagen I and (**f**) collagen II as observed by brown staining. Scale bar, 250 μm. The presence of collagen II was detected by 48 h, which is observed as light brown staining and indicated by the black arrows (80x magnification; scale bar, 25 μm). Representative images are shown of the 4–8 mice per group per time point.

To determine if PMMA bead injections could induce the deposition of collagen as a measure of fibrotic encapsulation, a Masson’s Trichrome stain was used to establish the presence of collagen as detected by blue staining. By day 7, collagen was detected amongst the beads within the sectioned aggregate (Fig. 2a) and by day 14, collagen was also detected surrounding the beads (Fig. 2b, blue layer). Within these sections, the presence of blood vessels and red blood cells were found at day 7 (black arrows, Fig. 2c) and day 14 (black arrows, Fig. 2d), indicating nascent vascularisation. Furthermore, at day 14, blue collagen staining was localised around individual PMMA beads at (white arrows, Fig. 2d).

To determine the type of collagen contributing to the encapsulating layer surrounding the PMMA bead aggregates, fibroblast-mediated collagen I deposition was probed because it is associated with tissue repair and is the most commonly reported type within implant-induced fibrotic capsules^13^,^14^,^15^. There was no collagen I detected within aggregate sections at any time point as shown by the lack of brown staining (Fig. 2e). Therefore, collagen II, which is secreted by chondrocytes^16^, was then investigated as an alternative source of collagen production. Deposition of collagen II was observed from 48 h within aggregate sections (black arrows, Fig. 2f), and by days 7 and 14, collagen II was in abundance surrounding the beads. Together, these results suggest that injections of PMMA bead into the peritoneal cavity induces a FBR, and thereby represents an ideal model for assessing the role of the inflammasome in this response *in vivo.*

### PMMA bead-mediated innate immune cellular infiltration is modulated by the ASC-dependent inflammasome

**Figure 3:**
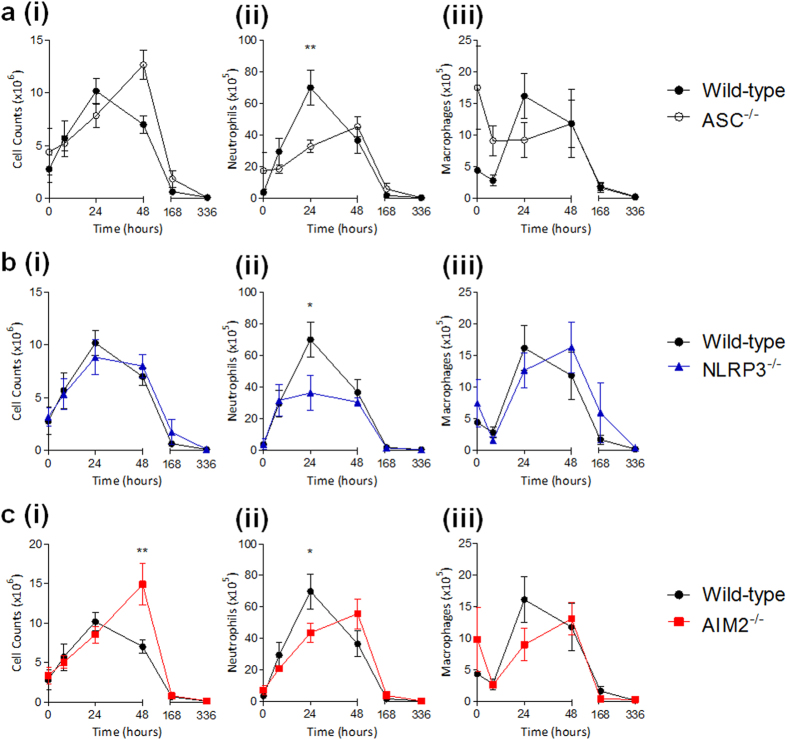
PMMA bead-mediated innate immune cellular infiltration is modulated by the ASC-dependent inflammasome. PMMA beads (200 μg/mouse) were injected i.p into (**a**) ASC-deficient mice (ASC^−/−^), (**b**) NLRP3-deficient mice (NLRP3^−/−^) or (**c**) AIM2-deficient mice (AIM2^−/−^) for the detection of (i) total cell numbers in the peritoneal exudates, and the absolute numbers of (ii) neutrophils and (iii) macrophages within the inflammatory period. Wild-type mice are shown for comparison. n = 4–8 mice/group. The mean ± SEM are shown. P values were calculated and are denoted as *P < 0.05 and **P < 0.01 as deduced via two-way analysis of variance.

To determine the effect of the inflammasome on the FBR, mice deficient in the common inflammasome mediator, ASC, were subjected to the adapted model of PMMA bead-induced FBR. We hypothesised that ASC^−/−^ mice would have reduced cell infiltrates and lower numbers of macrophages and neutrophils. Interestingly, ASC^−/−^ mice exhibited an increase in total cell infiltrate in peritoneal exudates, with the highest number observed at 48 h (12.7 ± 1.4 × 10^6^ cells), which was an 80% increase from wild-type mice (Fig. 3a(i)). However, in support of the original hypothesis, neutrophil levels were consistently lower and although ASC^−/−^ mice and wild-type mice had similar numbers at 48 h, neutrophil numbers were significantly reduced by 47% at 24 h during peak infiltration for wild-type mice (Fig. 3a(ii)). Macrophage numbers displayed different infiltration patterns to neutrophils because macrophages for ASC^−/−^ mice were at 17.4 ± 6.6 × 10^5^ cells in control mice (0 hr), which was almost 400% greater than macrophage numbers in wild-type mice (Fig. 3a(iii)). Despite an initial drop within 8 h, macrophage numbers in ASC^−/−^ mice did not seem to fluctuate and were equivalent to macrophage numbers in wild-type mice by 48 h (Fig. 3a(iii)).

To determine the inflammasome sensor responsible for these observations, the role of NLRP3 was investigated because it is known to be activated in biomaterial models both *in vivo* and *in vitro*^12^,^17^. Total cell numbers in peritoneal exudates upon PMMA bead injections were relatively consistent amongst NLRP3^−/−^ mice and wild-type mice (Fig. 3b(i)). Neutrophil numbers were, however, significantly decreased by 51% at 24 h when compared to wild-type mice (Fig. 3b(ii)). In contrast, macrophages in NLRP3^−/−^ mice were mostly consistent with wild-type mice, and were able to reach equivalent maximum numbers of 16.2 ± 4.0 × 10^5^ cells, however this was observed at 48 h (Fig. 3b(iii)).

The dispensable role of NLRP3 in optimal macrophage recruitment led us to explore the AIM2 sensor, which is an inflammasome sensor that is not a member of the NLR subfamily. AIM2 is responsible for detecting extracellular DNA and its relevance to the FBR may be associated with the detection of self DNA in phagocytes that are removing biomaterial-adherent apoptotic cells. It was found that PMMA bead injection into AIM2^−/−^ mice (Fig. 3c(i)) could induce cell infiltration in a similar manner to ASC^−/−^ mice (Fig. 3a(i)), whereby maximum infiltration was observed at 48 h with 14.9 ± 2.6 × 10^6^ cells, which was a 110% increase from wild-type mice (Fig. 3c(i)). Neutrophil numbers in AIM2^−/−^ mice were significantly reduced by 38% at 24 h when compared to wild-type mice, and peaked at 48 h with 55.6 ± 9.5 × 10^5^ cells (Fig. 3c(ii)). Macrophages in AIM2^−/−^ mice fluctuated with high starting numbers of 9.9 ± 5.1 × 10^5^ cells, which is double that of wild-type mice (Fig. 3c(iii)). Macrophage numbers of 13.1 ± 2.6 × 10^5^ cells in AIM2^−/−^ mice were comparable with wild-type at 48 h (Fig. 3c(iii)), which is in a similar manner to macrophage patterns for ASC^−/−^ mice (Fig. 3a(iii)).

### The ASC-dependent inflammasome does not influence deposition of albumin and fibrinogen

**Figure 4:**
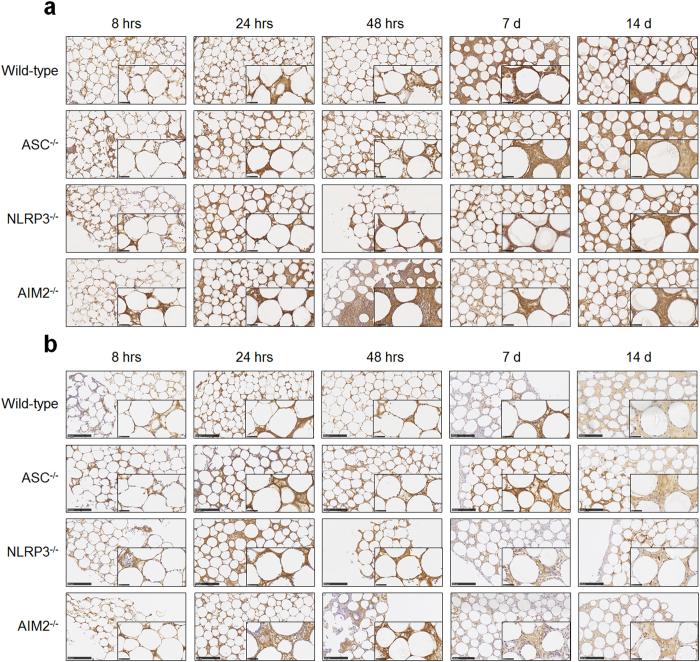
The ASC-dependent inflammasome does not influence deposition of albumin and fibrinogen. PMMA bead aggregates from ASC^−/−^, NLRP3^−/−^ or AIM2^−/−^ mice were sectioned at 5 μm for immunohistochemistry staining of (**a**) albumin and (**b**) fibrinogen as observed by brown staining. Wild-type mice are shown for comparison. Scale bar, 250 μm. Representative images are shown of the 4–8 mice per group per time point.

The adsorption of serum proteins was assessed, there were no obvious gross differences in the deposition of albumin (Fig. 4a) or fibrinogen (Fig. 4b) amongst sectioned bead aggregates from the different mice strains (wild-type is shown for comparison).

### Inflammasome components ASC and AIM2 but not NLRP3 modulate the progression of fibrotic encapsulation of injected PMMA beads

**Figure 5:**
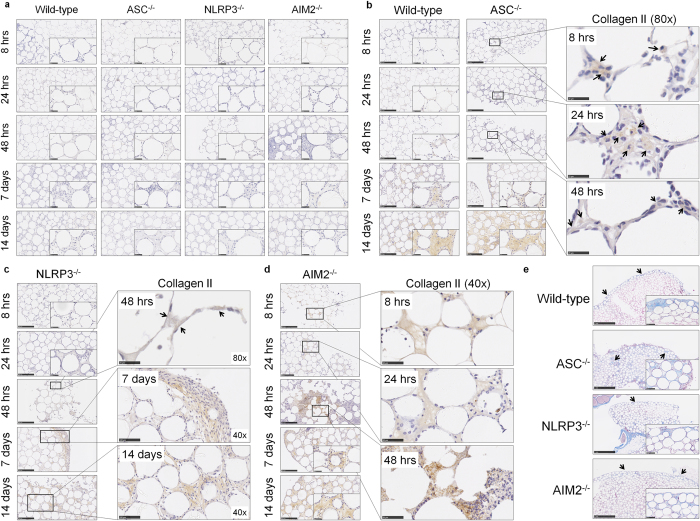
Inflammasome components ASC and AIM2 but not NLRP3 modulate the progression of fibrotic encapsulation of injected PMMA beads. (**a**) PMMA bead aggregates from ASC^−/−^, NLRP3^−/−^ or AIM2^−/−^ mice were sectioned at 5 μm for immunohistochemistry staining of collagen I. There was no collagen I detected as because no brown staining was observed. Sections from (**b**) ASC, (**c**) NLRP3, or (**d**) AIM2 deficient mice were stained for collagen II and observed as brown staining indicated by black arrows. (**e**) PMMA bead aggregates from ASC^−/−^, NLRP3^−/−^ or AIM2^−/−^ mice at day 14 were sectioned at 5 μm and stained with Masson’s Trichrome for detection of encapsulation. Wild-type mice are shown for comparison in (**b**) and (**e**). Representative images are shown of the 4–8 mice per group per time point.

The contribution of inflammasome components ASC, NLRP3 and AIM2 to fibrotic encapsulation was investigated by assessing collagen deposition using IHC. Collagen I was not detected in mice deficient in ASC, NLRP3 or AIM2 (Fig. 5a), however, collagen II was present in all of the samples (Fig. 5b–d). Collagen II deposition in aggregates from ASC^−/−^ mice exhibited premature recruitment of ‘collagen II positive’ cells within 8 h as observed by brown collagen staining localised in the cytoplasm (black arrows, Fig. 5b). Collagen II positive cells were also observed at 24 h, and deposition amongst the beads was detected at 48 h and day 7 (black arrows, Fig. 5b) before being in abundance by day 14 (Fig. 5b).

The absence of NLRP3 did not alter collagen II deposition when compared to wild-type (black arrows, Fig. 5c). However, when assessing collagen II staining in aggregates from AIM2^−/−^ mice (Fig. 5d), premature deposition was observed within 8 h that resembled the patterns of staining seen in ASC^−/−^ mice (Fig. 5b).

Aggregate sections stained with Masson’s Trichrome (Fig. 5e) revealed distinct blue staining for ASC^−/−^ and NLRP3^−/−^ mice amongst the beads, whereas there was a layer of blue stained collagen surrounding the beads in aggregates from AIM2^−/−^ mice (Fig. 5e).

### The composition of the PMMA bead-mediated cellular infiltrate is modulated by the ASC-dependent inflammasome

**Figure 6:**
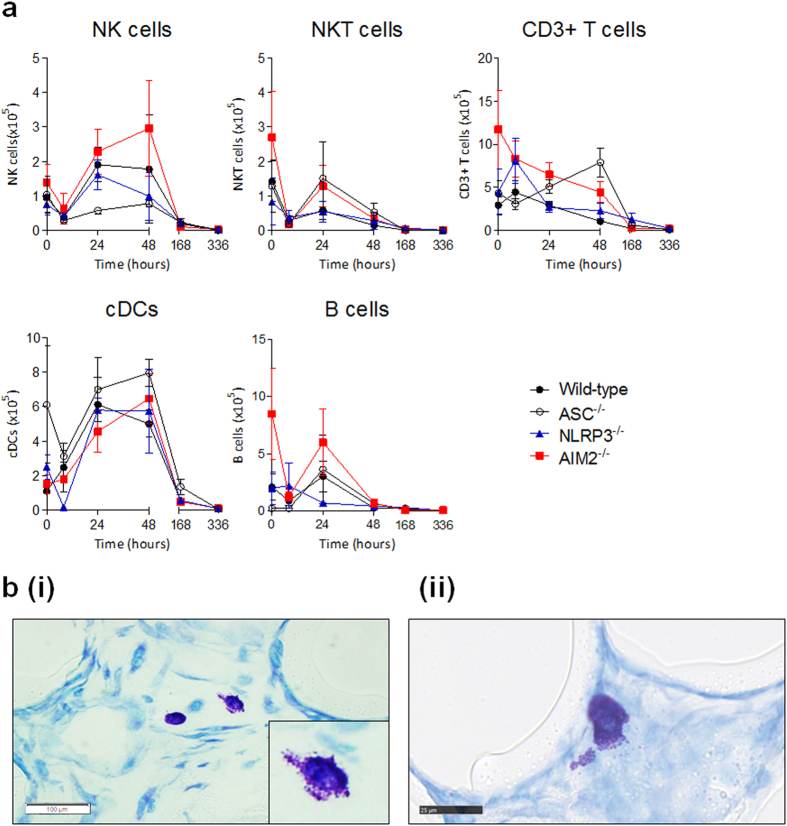
The composition of PMMA bead-mediated cellular infiltrate is modulated by components of the ASC-dependent inflammasome. (**a**) The peritoneal exudate of mice injected with PMMA beads (200 μg/mouse) were stained for the quantification of total cells numbers of NK cells (CD3^−^NK1.1^+^), NKT cells (CD3^+^ NK1.1^+^), CD3^+^ T cells (CD3^+^ NK1.1^−^) cDCs (CD11b^+^ CD11c^+^), and B cells (B220^+^ CD19^+^) using flow cytometry. n = 4–8 mice/group. The mean ± SEM are shown. P values were calculated and no significance was detected as deduced via two-way analysis of variance. (**b**) PMMA bead aggregates were sectioned at 4 μm and stained with toluidine blue for the detection of mast cells. Images are representative of aggregates from wild-type mice, and highlight the (i) presence of mast cells (scale bar, 100 μm) and (ii) degranulating mast cells (scale bar, 25 μm).

The presence of NK cells, NKT cells, CD3^+^ T cells, cDCs and B cells was measured in the peritoneal exudate of mice upon PMMA bead injections (Fig. 6a). In peritoneal exudates of wild-type mice, the NK cell, cDC and B cell numbers peaked at 24 h with totals of 1.9 ± 0.5 × 10^5^, 6.1 ± 1.6 × 10^5^ and 3.0 ± 1.3 × 10^5^ cells, respectively and gradually declined over time (Fig. 6a). There was a small increase in CD3^+^ T cells numbers from 2.9 ± 1.0 × 10^5^ cells at the 0 hr control time point to 4.4 ± 1.3 × 10^5^ cells at 8 h, before a steady decline over time. The NKT cells showed a different pattern because they were highest at 0 hr with a total of 1.4 ± 0.6 × 10^5^ cells but had dropped to 0.3 ± 0.1 × 10^5^ cells by 8 h, and this value remained consistent over time (Fig. 6a).

In ASC^−/−^ mice, B cell numbers were consistent with numbers in wild-type mice, however the remaining cell types were modulated. NK cells were markedly reduced in the absence of ASC signalling, with numbers on average, 63% lower than that of wild-type mice (Fig. 6a). Upon PMMA bead injections, the highest NK cell number was observed at 48 h, with 0.8 ± 0.1 × 10^5^ cells compared to wild-type mice values of 1.8 ± 1.6 × 10^5^ cells at the same time point. In contrast, NKT cells were at higher values with 1.5 ± 1.1 × 10^5^ cells within 24 h, which was 158% greater than wild-type mice. The numbers of CD3^+^ T cells in ASC^−/−^ mice were also higher than that of wild-type mice, as they gradually increased to peak at 48 h with 7.9 ± 1.6 × 10^5^ cells compared to 1 × 10^5^ cells in wild-type mice. The cDCs in ASC^−/−^ mice were at starting populations of 6.1 ± 3.4 × 10^5^ cells at 0 h, whereas cDC numbers in wild-type mice begun at 1.1 ± 0.1 × 10^5^ cells. Despite a drop in numbers in 8 h, cDCs in ASC^−/−^ mice rebounded by 24 h, and continued to increase to 8.0 ± 0.8 × 10^5^ cells at 48 h in contrast to cDCs in wild-type mice were beginning to decline (Fig. 6a).

In NLRP3^−/−^ mice, NKT cell and cDC numbers were equivalent to wild-type mice (Fig. 6a). Although CD3^+^ T cells were relatively consistent with wild-type mice, there was a small peak at 8 h that reached 8.1 ± 2.6 × 10^5^ cells, which was 82% higher than wild-type numbers. On the other hand, NK cells were consistent with wild-type until 48 h where we observed a 45% drop in numbers to 1.0 ± 0.7 × 10^5^ cells in NLRP3^−/−^ mice. Similarly, we observed a 77% decrease in B cells number at 24 h, however B cells remained relatively consistent with wild-type for the remainder of the inflammatory period (Fig. 6b).

The AIM2^−/−^ mice demonstrated the greatest variation in leukocyte numbers as compared to wild-type mice, with the exception of cDCs, which were at equivalent numbers of 6.5 ± 1.4 × 10^5^ cells at 48 h despite these peak numbers being delayed from wild-type mice by 24 h (Fig. 6a). Numbers of NK cells in AIM2^−/−^ mice were on average 44% higher than that of wild-type mice, and NK cells peaked at 48 h with 3.0 ± 1.4 × 10^5^ cells. Not only were NK cells higher wild-type mice, but were on average 279% greater than NK cell numbers in ASC^−/−^ mice, suggesting a more pronounced role of AIM2 in regulating NK cells. In contrast, NKT cells were equivalent amongst AIM2^−/−^ and ASC^−/−^ mice, and were greater than that of wild-type mice, notably at 24 h with a peak of 1.3 ± 0.6 × 10^5^ cells. The CD3^+^ T cell numbers were initially higher in AIM2^−/−^ mice, being 300% greater than wild-type mice with starting numbers of 11.7 ± 4.6 × 10^5^ cells, however this steadily declined over the inflammatory period. The numbers of B cells were also 300% higher than that of wild-type with 0 hr control mice having 8.5 ± 4.0 × 10^5^ cells B cells, before dropping at 8 h, and regaining numbers to 6.0 ± 3.0 × 10^5^ cells at 24 h. This peak value during the inflammatory period was double that of the B cells in wild-type mice (Fig. 6a).

Another leukocyte subset involved in the FBR, mast cells, were assessed because they are consistently reported at the site of implantation^18^,^19^,^20^,^21^, and can be a source of IL-4 and IL-13 that drive a ‘wound healing’ phenotype in macrophages that result in the secretion of pro-fibrogenic factors. Mast cells were detected by staining aggregate sections with toluidine blue (Fig. 6b), and were found in all samples from as early as 8 h and throughout the inflammatory period. Representative images highlight the presence of mast cells amongst the beads (Fig. 6b(i)), and we observed the degranulation of mast cells (Fig. 6b(ii)), which appeared to be interacting with the surface of the bead, suggesting that these recruited mast cells were also activated.

### Inflammasome components ASC and AIM2 modulated the cytokine milieu

**Figure 7:**
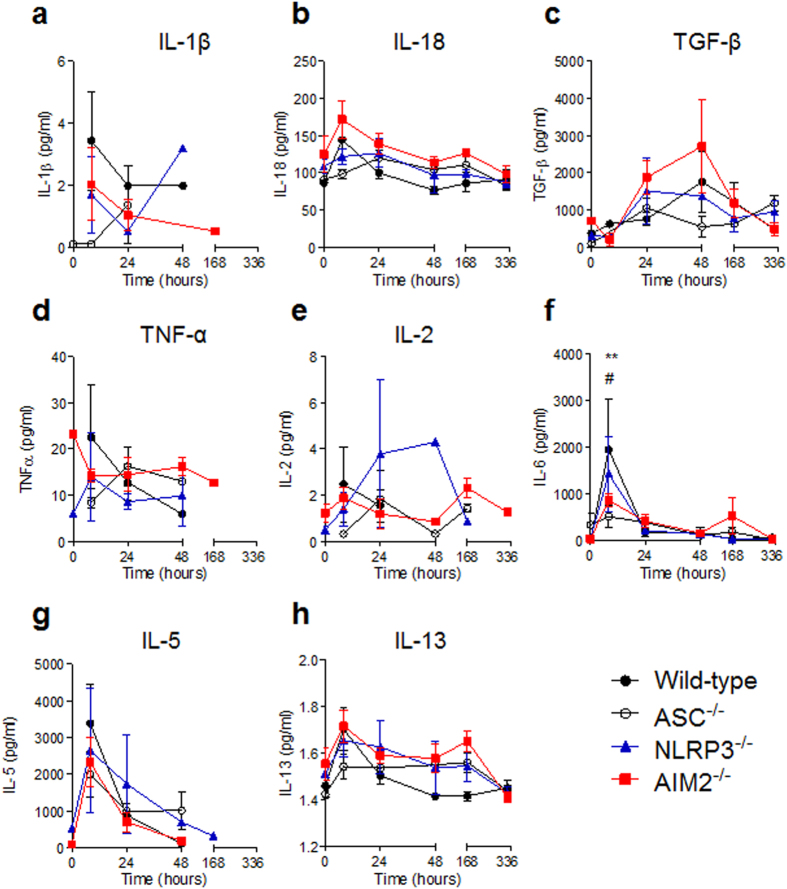
Cytokine composition in the peritoneal exudate of mice injected with PMMA beads. The peritoneal exudate of mice injected with PMMA beads (200 μg/mouse) was collected via lavage for the assessment of cytokine concentrations using the MAGPIX assay. (**a**) IL-1β, (**b**) IL-18, (**c**) TGF-β, (**d**) TNF-α, (**e**) IL-2, (**f**) IL-6, (**g**) IL-5 and (**h**) IL-13 levels were quantified. n = 2–8 mice/group. The mean ± SEM are shown. P values were calculated using a two-way analysis of variance with a Dunnett’s post-test to compare each genotype against wild-type mice. ASC^−/−^ mice, **P < 0.01; and AIM2^−/−^ mice, ^#^P < 0.05.

The altered leukocyte profiles in mice deficient in inflammasome components led to the assessment of inflammatory cytokines within the peritoneum. Given that the responses of NLRP3^−/−^ mice were more consistent with wild-type mice, we hypothesised that the NLRP3 inflammasome was dispensable; however ASC^−/−^ and AIM2^−/−^ mice would have reduced levels of IL-1β and IL-18 in the peritoneal exudates. Wild-type mice receiving PMMA bead injections revealed maximum IL-1β levels of 3.4 pg/ml at 8 h, with a reduction to 2.0 pg/ml by 24 h that remained consistent at 48 h (Fig. 7a). Consistent with the hypothesis, ASC^−/−^ mice had lower concentrations of IL-1β until 1.3 pg/ml was detected at 24 h. Interestingly, when compared to wild-type mice, NLRP3^−/−^ mice had an approximate 50% and 400% reduction of IL-1β at 8 h and 24 h respectively, before an increase to 3.2 pg/ml at 48 h. As hypothesised, IL-1β levels were reduced for AIM2^−/−^ mice, however despite undetectable levels at 48 h, IL-1β was detected at day 7 (Fig. 7a). Unexpectedly, IL-18 levels were varied and in fact, raised for AIM2^−/−^ mice throughout the inflammatory period above that of wild-type mice (Fig. 7b). At 0 h, AIM2^−/−^ mice had 38% greater levels of IL-18 compared to wild-type mice, however both genotypes had peak concentrations at 8 h with 171.8 pg/ml and 144.3 pg/ml, respectively. Alternatively, ASC^−/−^ and NLRP3^−/−^ mice had peak IL-18 levels at 24 h with 119.7 pg/ml and 126.3 pg/ml being detected. Of note, all mice deficient in inflammasome components had higher and persistent levels of IL-18 from 24 h post-injection of PMMA beads, when compared to wild-type mice (Fig. 7b).

The observation that ASC^−/−^ and AIM2^−/−^ mice had premature collagen II deposition led to the hypothesis that TGF-β1 concentration may be elevated in these mice. Collagen II is exclusively secreted by chondrocytes^16^, and it has been shown that TGF-β induced collagen II synthesis is reduced in the presence of IL-1β^22^,^23^. In support of this hypothesis, it was found that TGF-β1 was elevated by 250% and 55% for AIM2^−/−^ mice at 24 and 48 h, respectively, when compared to wild-type mice (Fig. 7c). In contrast, ASC^−/−^ mice had reduced levels of TGF-β1, whereas NLRP3^−/−^ mice had peak levels of 1504.5 pg/ml at 24 h, which was slightly lower than maximal levels in wild-type mice of 1755.1 pg/ml at 48 h (Fig. 7c).

Next, pro-inflammatory cytokines TNF-α (Fig. 7d), IL-2 (Fig. 7e) and IL-6 (Fig. 7f) were assessed and also found to vary depending on the genotype. Unexpectedly, AIM2^−/−^ and NLRP3^−/−^ mice had baseline readings of TNF-α of 23.2 pg/ml and 5.9 pg/ml at 0 h (Fig. 7d). Wild-type mice displayed peak TNF-α of 22.6 pg/ml at 8 h, whereas ASC^−/−^ mice reached peak TNF-α levels of 16.2 pg/ml at 24 h. Despite a slight drop at 8 h, TNF-α was maintained up to day 7 in AIM2^−/−^ mice (Fig. 7d), and the only genotype to maintain IL-2 up to day 14 (Fig. 7e). The secretion of IL-2 from ASC^−/−^ mice was mostly consistent with AIM2^−/−^ mice except for low starting levels at 8 h. Most strikingly was the high levels of IL-2 for NLRP3^−/−^ mice at 24 h and 48 h, with concentrations of 3.8 pg/ml and 4.3 pg/ml, respectively, when compared to wild-type mice levels of 1.5 pg/ml at 24 h and undetectable concentrations at 48 h (Fig. 7e). In the case of IL-6, maximum levels were detected at 8 h for wild-type mice, which was statistically greater than ASC^−/−^ and AIM2^−/−^ mice (Fig. 7f). ASC^−/−^ mice had the lowest IL-6 levels throughout the inflammatory period, followed by AIM2^−/−^ mice, however despite lower than maximum, 521.7 pg/ml of IL-6 was detected at day 7, which was 27 times greater than that of wild-type (Fig. 7f).

When IL-5 was assessed, all murine genotypes appeared to follow a similar trend despite lower concentrations for mice deficient in inflammasome components when compared to wild-type mice (Fig. 7g). Notably, IL-5 was most persistent in NLRP3^−/−^ mice, being detected at a low concentration at day 7 (Fig. 7g). In the case of IL-13, maximum levels in wild-type mice were observed at 8 h before returning to baseline concentrations at 24 h (Fig. 7h). Differences were observed for NLRP3^−/−^ and AIM2^−/−^ mice from 24 h, which maintained their IL-13 levels throughout the inflammatory period. This was also observed for ASC^−/−^ mice except that there was overall reduction from as early as 8 h (Fig. 7h). Nonetheless, IL-13 levels were comparable by day 14 regardless of murine genotype, and in agreeance with the detection of mast cells, most commonly described to be the source of IL-13 in the FBR^24^,^25^.

## Discussion

Unravelling the contribution of inflammasome components on a biomaterial-induced FBR may direct the development of targeted therapies that reduce the negative outcomes of implantation. In the pursuit of characterising the inflammatory reaction of the FBR, we first aimed to adapt and further develop a robust *in vivo* model that would negate the complications of surgical implantation, but was able to develop the full features of the FBR. To this end, we revised a recently published *in vivo* model of injecting PMMA beads of 150 μm in diameter, and classified the host response over several time points to demonstrate the development of the FBR. A key new observation from our results was the aggregation of the bead suspension into a solid mass *in vivo*, and as early as 8 h post-injection, which had not been previously reported^26^. Importantly, this allowed the investigation of the protein composition surrounding the beads that contributed to their aggregation, particularly in the chronic phase during fibrotic encapsulation. Despite collagen I being the major type found in fibrous tissue^27^,^28^,^29^, the lack of detectable collagen I may have been due to the relatively short time frame of the experiments and tissue location of the bead injection. However, the presence of collagen II, which is exclusively secreted by chondrocytes found in cartilage^16^, was surprising. Therefore, we speculate that the source of chondrocytes in this model are mesenchymal stem cells (MSCs), which we propose are recruited to the site of injury and differentiate into chondrocytes^30^ through the action of TGF-β^31^,^32^,^33^,^34^,^35^.

When IL-1β expression was assessed, the reduced levels in mice deficient in any of the inflammasome components suggest the involvement of the NLRP3 and AIM2 inflammasome to some, albeit, differing extents. However, based on the protein and cellular inconsistencies between ASC^−/−^, NLRP3^−/−^ and AIM2^−/−^ mice, the results would also indicate that these proteins may play inflammasome-independent roles within this model of the FBR. The most pronounced discrepancy was the premature collagen II deposition in ASC^−/−^ and AIM2^−/−^ mice; however these genotypes presented varied leukocyte and cytokine profiles. In particular, the higher levels of TGF-β1 and lower concentrations of IL-1β in AIM2^−/−^ mice compared to wild-type mice strengthens the hypothesis of MSC differentiation into chondrocytes because it has been shown that TGF-β-induced collagen II synthesis is reduced in the presence of IL-1β^22^,^23^. Although causative conclusions cannot be made in this study, AIM2^−/−^ mice also presented high numbers of NK cells and Almeida *et al.* (2011) found that NK cells could recruit but not activate MSCs^36^. Therefore, our results may suggest that NK cells can actively recruit MSCs that lead to TGF-β1 driven differentiation and collagen II secretion, which is encouraged in an environment of reduced IL-1β levels in AIM2^−/−^ mice. Recent studies have also demonstrated inflammasome-independent roles for AIM2, primarily for the protection against colon cancer^37^,^38^ by acting to inhibiting the Akt kinase involved in cell proliferation and survival^37^. Interestingly, the Akt pathway is implemented in regulating collagen production^39^,^40^,^41^, MSC differentiation into chondrocytes^42^, and in fact is activated upon TGF-β1 signalling through its receptors on chondrocytes^43^. Taken together, we propose that an AIM2 deficiency is activating Akt pathways, thereby promoting chondrocyte secretion of collagen II through TGF-β1 signalling, as well as enhanced cell proliferation to explain the higher number of leukocytes in these mice. It would be of value to assess the effects of Akt inhibitors on the fibrotic encapsulation of biomaterials in this model.

However, the demonstration of the AIM2 inflammasome involvement in this model then suggests that the AIM2 sensor protein is being engaged, which we believe to be the case. We suggest two possible sources of self dsDNA for recognition by and activation of AIM2. Firstly, AIM2 has demonstrated the capacity to recognise and activate upon mitochondrial DNA binding, in both oxidized and non-oxidized forms *in vitro*^44^. Therefore, we suggest that excessive ROS in macrophages and neutrophils^5^,^45^ can cause mitochondrial damage and induce apoptotic pathways^46^. Secondly, extracellular DNA from NETs^47^,^48^ were shown to be recognised by TLR-9, therefore, we propose that a second source of self dsDNA for AIM2 recognition may be through the formation of NETs in a model of biomaterial-induced inflammation^47^,^48^. A recent report provided further rationale for NET involved in biomaterial-induced inflammation. Branzk *et al.* (2014) demonstrated that neutrophils differentially respond to yeast morphology; yeast that were small enough to be engulfed could be cleared via intracellular killing mechanisms, whereas the hyphae form selectively released NETs from neutrophils^49^. This presents a novel avenue for exploring differential neutrophil responses against macroscopic biomaterial implants, and specifically the involvement of DNA sensors in this process.

In the case of the NLRP3 inflammasome, it did not appear to be involved in biomaterial-induced FBR in our model despite being previously implicated in responding to foreign non-phagocytosable particulates^7^,^8^,^9^,^50^,^51^ Despite acute perturbations in NLRP3^−/−^ mice, both macrophage number and IL-1β concentrations were comparable, albiet delayed, to wild-type mice, consistent with the observations of Malik *et al.* (2011) suggesting that NLRP3 is dispensable in the chronic process^12^. These results reiterate that the correlation between NLRP3 activation and biomaterial-induced inflammation remains to be fully explained. Triggering NLRP3 activation by non-phagocytosable particles has been implicated via direct membrane ligation, which is independent of lysosomal or phagosomal pathways^52^. One emerging theory for NLRP3 activation by non-phagosomal mechanisms is membrane affinity triggered signalling (MATS), which describes perturbation of lipid rafts in the cell membrane that can activate Syk kinase, by clustering receptors that contain immunoreceptor tyrosine-based activation motifs^53^. In the case of MSU crystals, it has been suggested that direct interactions with a cell can alter the membrane curvature and result in the opening of ion channels^52^. It is also known that K^+^ efflux is one model of NLRP3 activation, and was recently found to be the common outcome amongst NLRP3 stimuli^54^. Although a defect in the K^+^ efflux within PMMA bead-bound leukocytes in NLPR3-deficient mice was not demonstrated in this study, the MATS model does present a plausible explanation for the results because adherent cells were observed alter their cytoskeletal shape. This, in addition to the curvature of the individual bead, may cause membrane tension and induce mechanosensing pathways to trigger NLRP3 activation. Additionally, the assumption that MATS is occurring in this model may justify the discrepancies of acute and chronic responses; early responses may involve NLRP3 as the inflammatory response is developing, however in the absence of TLR ligation or ‘signal 1’ due to the sterile environment, the resulting NFκB activation is not induced, and cannot transcriptionally control NLRP3 expression to potentiate intracellular sensing^55^,^56^.

In summary, we present an *in vivo* model of peritoneal FBR using PMMA bead injections to assess the role of the inflammasome in biomaterial-induced inflammatory pathways. Our results support the involvement of the ASC-dependent inflammasome in biomaterial-induced FBR and revealed a potential inflammasome-independent role of AIM2 in collagen production and leukocyte recruitment. Together, we anticipate that further investigation into the exact mechanisms of how inflammasome components regulate FBR processes will allow the identification of targetable pathways for therapeutic intervention to enhance the utility of biomaterial implantation.

## Methods

### Animals

All experimental protocols were approved by the Animal Ethics Committees of SA Pathology (Project Number 56.12) and the University of Adelaide (Project Number M-2012-11). All methods were performed in accordance to the guidelines of the University of South Australia (South Australian Animal Welfare Act 1985). Wild-type C57Bl/6 (B6) mice were purchased from Laboratory Animal Services (University of Adelaide, SA, Australia). Mice deficient in ASC were kindly provided by Professor Vishwa Dixit, Yale University. Mice deficient in NLRP3 were kindly provided by Professor Hal Hoffman, University of California, San Diego. Mice deficient in AIM2 were kindly provided in Professor Kate Fitzgerald, University of Massachusetts. Mice deficient in the inflammasome components were generated on a B6 background. Animals were housed in the Reid Animal Facility (University of South Australia) under pathogen-free conditions.

### Poly(methylmethacrylate) (PMMA) bead preparations

Poly(methylmethacrylate) (PMMA) non-functionalised beads (Bangs Labs, BB05N/5438) were obtained as dry powder of microspheres of 125–180 μm diameter with a mean diameter of 153 μm. The PMMA beads were extensively washed three times with sterile 70% ethanol, followed by four washes with sterile EF-PBS. Washes were performed by adding 10–15 ml of solution, gently rotating tube to ensure all beads were adequately exposed, and settled by centrifugation (flick spin to maximum speed) prior to removing solution and adding fresh solution. To ensure the sterility of PMMA bead preparations prior to implantation, we performed endotoxin testing using the Endosafe®-PTS kit (Charles River Laboratories, Wilmington, MA, USA) according to manufacturer’s instructions. The samples were below the limit of detection with readings under 0.100 EU/ml (1EU = 100 pg), qualifying the resultant inflammatory response *in vivo* to be independent of endotoxin contamination.

### Injection of PMMA beads

Prior to PMMA bead injection into the mice, the beads are prepared in an injection solution of EF-PBS containing 0.02% (vol/vol) ethanol and 0.02% (vol/vol) Pluronic F-127 (AAT Bioquest) to reduce bead-bead repulsion. Syringes (1 ml) were prepared by aspirating ~200 μg beads and topping up to a total of 500 μl of injection solution when a 21G needle was fitted. Syringe and needles were maintained upright or on their side to ensure the beads did not enter and clog the needle opening. The bevel of the needle was in line with the measurements on the barrel of the syringe.

Prior to injection, mice were briefly anaesthetised using isofluorane (2% in 1L/O_2_) during which the syringe was gently rotated to allow the beads to move through the solution and become more homogenous, ensuring the beads did not enter the needle opening. Mice were injected via the intraperitoneal (i.p) route, ensuring a gentle and steady rate of injection to prevent bead blockage of the needle. Experimental mice were observed to recover from anaesthetic and monitored for the remainder of the experiment (up to 14 days).

### Peritoneal lavages and immunophenotyping cell infiltrates

For the analysis of cell phenotypes at the injection site, peritoneal lavages were performed by gently injecting 1 mL of cold EF-PBS through a 21G needle and massaging the cavity to potentially dislodge any attached cells, before slowly retrieving the EF-PBS. A following two washes were performed with 2 mL of cold EF-PBS, and cells were pelleted from the total 5 mL exudate by centrifugation prior to red blood cell lysis of the resulting pellet. Cells were stained with the following surface antibodies for 30 mins on ice, washed, detected on the FACSCanto II flow cytometer and then analysed using FlowJo for the classification of: neutrophils (CD11b^+^ Gr-1^+^), macrophages (CD11b^+^ F4-80^+^), NK cells (CD3^−^NK1.1^+^), cDCs (CD11b^+^ CD11c^+^), NKT cells (CD3^+^ NK1.1^+^), CD3^+^ T cells (CD3^+^ NK1.1^−^) and B cells (B220^+^ CD19^+^). The gating strategy is represented in Supplementary Fig. S1.

### Staining PMMA bead-adherent leukocytes

Disaggregated PMMA beads were stained with Diff-Quik for the detection of adherent leukocytes as per manufacturer’s instructions. Briefly, beads were incubated with Solution A for 5 secs, before the solution was removed and Solution B added for an additional 10 secs. Beads were extensively washed with tap water and pipetted onto coverslips for imaging using the brightfield filter on an Olympus IX51 Fluorescence Microscope (Olympus) and captured via the cellSens program (Olympus).

### Quantifying inflammatory cytokines from peritoneal exudates

To determine the cytokine environment upon PMMA bead injection, the initial 1 mL used to wash the peritoneum was assessed used by first removing cells via centrifugation and then storing supernatant at −20 °C until use. Cytokine concentrations were measured using a ProcartaPlex multiplex kits (Jomar Life Research) detected on the Bio-Plex MAGPIX Multiplex Reader (Bio-Rad) according to manufacturer’s instructions. The Th1/Th2 multiplex kit was used for the detection of IL-1β, IL-2, IL-4, IL-5, IL-6, IL-12p70, IL-13, IL-18, TNF-α, IFNγ and GM-CSF, and combined with simplex kits for the detection of IL-1α and IL-10. The detection of TGF-β1 was perfomed using a simplex kit, however samples were prepared prior to assessment. Peritoneal exudates were acid treated using 1N HCl for 10 mins at RT, followed by neutralisation with a 1N NaOH/0.5M HEPES solution.

Samples were below the limit of detection for IL-1α, IL-10, IL-12p70, IFNγ and GM-CSF (data not shown) and in some cases, specific time points were below the limit of detection and omitted from the graphs represented in Fig. 7.

### Bead aggregates processing and sectioning

Bead aggregates were surgically removed from the peritoneum of mice, and immediately fixed in 1 ml of 10% neutral formalin buffer. Aggregates were subsequently transferred to histology cassettes and held in place with small foam squares. Cassettes were placed in 10% neutral formalin buffer and immediately processed on a short cycle in a Tissue Tek VIP 2000 tissue processor (GMI Inc. Ramsey, MN, USA) and subsequently embedded in paraffin wax for sectioning. Paraffin blocks were sectioned using a manual rotary microtome (Leica RM2235, Leica Camera, Wetzlar, HE, Germany) and placed on a cold plate set at 60 °C to remove moisture from the sections and allow stronger adhesion of the sample to the microscope slide. Sections of 5 μm were cut for immunohistochemistry and sections of 4 μm were cut for the detection of mast cells using toluidine blue staining. Prior to staining, paraffin block sections were de-waxed by immersion in xylene (2 mins with gentle agitation) and then hydrated with one wash of absolute ethanol followed by 90% ethanol for one minute with gentle agitation. Sections were placed in tap water prior to staining. All stained sections were imaged using the NanoZoomer NDPI system (Hamamatsu Photonics, Hamamatsu City, Shizuoka Pref, Japan) at 40x magnification with manual focussing, and images were analysed using the NDP.view 2 software (Hamamatsu Photonics).

### Immunohistochemistry and staining

The immunohistochemistry of albumin, fibrinogen and collagen I and collagen II were contracted to and performed by Dr Jim Manavis using standard procedures of the Centre for Neurological Diseases Laboratory.

### Mast cell staining

Mast cells were detected by toluidine blue stain as previously described^57^. Briefly, 1.0% aqueous toluidine blue (Sigma) was diluted 1:10 (v/v) with 1N HCl to prepare a 0.1% toluidine blue solution. The solution was generously pipetted over de-waxed and hydrated aggregate sections for 90 s before rinsing off with a gentle and steady stream of tap water. Sections were air-dried and coverslips mounted with DEPEX solution (Sigma).

### Statistical analysis

Two-way analysis of variance was performed with a Dunnett’s posttest for the statistical analysis of cell phenotypes, where wild-type B6 mice were the control group. Statistical significance was not observed unless otherwise stated.

## Additional Information

**How to cite this article**: Christo, S. N. *et al.* Inflammasome components ASC and AIM2 modulate the acute phase of biomaterial implant-induced foreign body responses. *Sci. Rep.* **6**, 20635; doi: 10.1038/srep20635 (2016).

## Supplementary Material

Supplementary Information
