# Supplementary material for: Inflammasome components ASC and AIM2 modulate the acute phase of biomaterial implant-induced foreign body responses
Source: Sci Rep. 2016 Feb 10;6:20635. doi: 10.1038/srep20635 (PMC4748295; doi:10.1038/srep20635)

SUPPLEMENTARY INFORMATION

Inflammasome components ASC and AIM2 modulate the acute phase of biomaterial implant-induced foreign body responses

Susan N. Christo, Kerrilyn R. Diener, Jim Manavis, Michele A. Grimbaldeston, Akash Bachhuka, Krasimir Vasilev, and John D. Hayball.

Supplementary Figure legends

Supplementary Fig. S1. Gating strategy for immunophenotyping leukocyte populations in the peritoneal exudates of mice receiving i.p PMMA bead injections. The peritoneal exudate of mice injected with PMMA beads (200 µg/mouse) was collected via lavage for the immunophenotyping of leukocyte populations. Cells were gated on a forward and side scatter population to exclude debris, followed by the classification of the following leukocytes: neutrophils (CD11b$^+$Gr-1$^+$), macrophages (CD11b$^+$F4-80$^+$), cDCs (CD11b$^+$CD11c$^+$), B cells (B220$^+$CD19$^+$), NK cells (CD3$^-$NK1.1$^+$), NKT cells (CD3$^+$NK1.1$^+$) and CD3$^+$ T cells (CD3$^+$NK1.1$^-$). Images are representative of data from wild-type mice at 24 hrs post-injection.

Supplementary Figures

Supplementary Fig. S1.

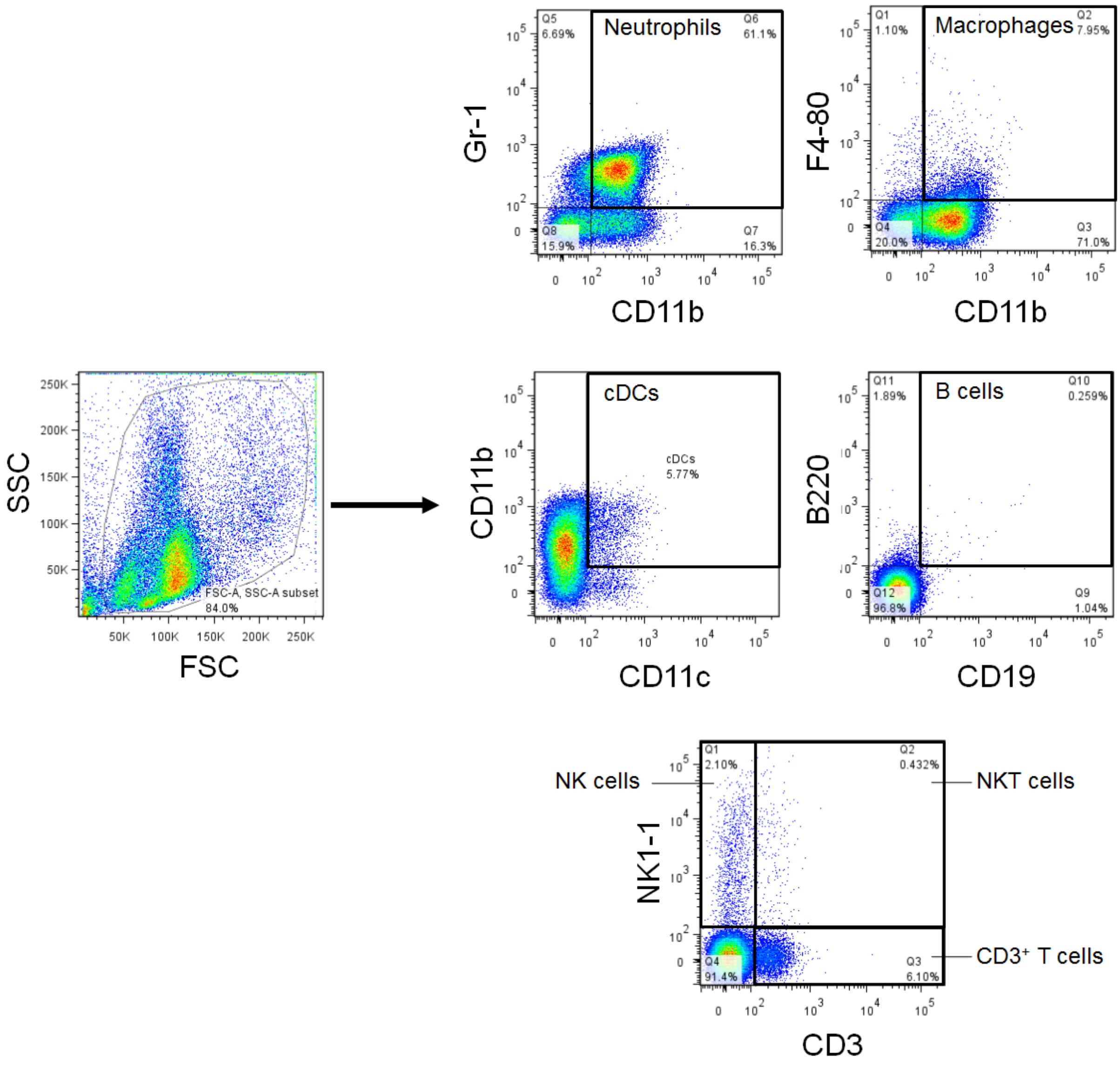

Supplement: Supplementary Information [file srep20635-s1.pdf]
